# Functional Expression of the Recombinant Spike Receptor Binding Domain of SARS-CoV-2 Omicron in the Periplasm of *Escherichia coli*

**DOI:** 10.3390/bioengineering9110670

**Published:** 2022-11-10

**Authors:** Woo Sung Kim, Ji Hyun Kim, Jisun Lee, Su Yeon Ka, Hee Do Chae, Inji Jung, Sang Taek Jung, Jung-Hyun Na

**Affiliations:** 1 Department of Pharmaceutical Engineering, Sangji University, Wonju 26339, Korea; 2 Department of Biomedical Sciences, Graduate School, Korea University, Seoul 02841, Korea; 3 BK21 Graduate Program, Department of Biomedical Sciences, Korea University College of Medicine, Seoul 02841, Korea; 4 Institute of Human Genetics, Korea University College of Medicine, Seoul 02841, Korea; 5 Research Institute of Korean Medicine, Sangji University, Wonju 26339, Korea

**Keywords:** spike receptor binding domain, omicron, functional expression, *E. coli*

## Abstract

A new severe acute respiratory syndrome coronavirus 2 (SARS-CoV-2) variant known as Omicron has caused a rapid increase in recent global patients with coronavirus infectious disease 2019 (COVID-19). To overcome the COVID-19 Omicron variant, production of a recombinant spike receptor binding domain (RBD) is vital for developing a subunit vaccine or a neutralizing antibody. Although bacterial expression has many advantages in the production of recombinant proteins, the spike RBD expressed in a bacterial system experiences a folding problem related to disulfide bond formation. In this study, the soluble Omicron RBD was obtained by a disulfide isomerase-assisted periplasmic expression system in *Escherichia coli*. The Omicron RBD purified from *E. coli* was very well recognized by anti-SARS-CoV-2 antibodies, sotrovimab (S309), and CR3022, which were previously reported to bind to various SARS-CoV-2 variants. In addition, the kinetic parameters of the purified Omicron RBD upon binding to the human angiotensin-converting enzyme 2 (ACE2) were similar to those of the Omicron RBD produced in the mammalian expression system. These results suggest that an *E. coli* expression system would be suitable to produce functional and correctly folded spike RBDs of the next emerging SARS-CoV-2 variants quickly and inexpensively.

## 1. Introduction

COVID-19 has threatened global human public health and the economy since December 2019. Because older adults or people with medical complications are more likely to have a poor prognosis among SARS-CoV-2-infected people [1,2], many countries have been imposing strict social distancing measures, tightening public hygiene, and encouraging vaccinations. Although 63% of the world population has been fully vaccinated [3], about 620 million cumulative COVID-19 cases have been confirmed [4,5], including 6.5 million deaths worldwide [5,6] according to the statistics of the World Health Organization.

The steady global increment of COVID-19 cases has caused the rapid emergence of SARS-CoV-2 variants. In particular, the Alpha (B.1.1.7), Delta (B.1.617.2), and Omicron (BA.1, BA.2, BA.2.12.1, BA.4, and BA.5) variants of SARS-CoV-2 have been prevalent and have produced peaks in COVID-19 cases [7,8]. Among these variants, Omicron has become the latest dominant strain of COVID-19 worldwide [9] after it was first reported in South Africa (BA.1). Currently, several subvariants (BA.2, BA.2.12.1, BA.4, and BA.5) have emerged [10]. The efficacy and protection of US FDA-approved COVID-19 vaccines against diseases caused by Omicron were significantly lower than against previous SARS-CoV-2 variants [11], and Omicron presented a higher reinfection rate compared to other SARS-CoV-2 variants [12]. Additionally, FDA-approved therapeutic antibodies for COVID-19 showed significantly lower neutralizing efficacy against Omicron [13,14,15], indicating the necessity for vaccines and therapeutics specially designed for the Omicron variant. Along this line, the recombinant spike RBD of SARS-CoV-2 is a prerequisite material for developing vaccines and neutralizing antibodies against COVID-19 because the entry of SARS-CoV-2 into the host cell depends on the viral spike protein that binds to the human angiotensin-converting enzyme 2 (ACE2) [16]. To generate therapeutic antibodies with potent neutralization activity, most therapeutic anti-SARS-CoV-2 antibodies have targeted the spike RBD, and many subunit vaccines have been developed based on the recombinant RBD region [16]. Therefore, it is of great importance to develop an excellent preparative method for the recombinant spike RBD of SARS-CoV-2.

Compared with other expression hosts, *E. coli* has many advantages for heterologous recombinant protein production in terms of growth speed, productivity, cost, and ease of scale-up [17]. Despite these merits, an *E. coli* expression system is not preferred in the production of a recombinant spike RBD, with four disulfide bonds [18] that are not correctly folded in the cytoplasm of *E. coli,* which is maintained in a reduced condition. Some researchers have tried to solve the folding problem by refolding the RBD [19,20,21,22,23], but the biochemical properties of the refolded RBD were quite different from those of the RBDs expressed in insect or mammalian cell systems [22]. The Borgstahl lab reported a soluble expression method of the recombinant RBD in the cytoplasm of *E. coli* using the enzyme (Evr1p and PDI)-assisted co-expression system CyDisCo [24,25]. However, the recombinant RBD co-expressed with the CyDisCo has a lower ACE2 binding affinity than the RBD expressed in the insect cell system, and there was no cleavage data of the maltose binding protein (MBP) tag [24], indicating that the untagged RBD might be insoluble [26] or have different biophysical properties compared to the RBD expressed in an insect or mammalian cell system. The Tsumoto lab also reported soluble RBDs of SARS-CoV-2 wild type and Omicron without an MBP tag expressed in *Brevibacillus choshinensis,* a gram-positive bacterium. However, there were no ACE2 binding data [26], leaving unclear the biochemical properties of the recombinant proteins.

To solve these issues of the bacterial expression system, we used an enzyme-assisted periplasmic *E. coli* expression system for producing the spike RBD of the SARS-CoV-2 Omicron variant. Owing to the existence of disulfide bond-catalyzing chaperones in the periplasm of *E. coli* [27], secretion into the periplasm is a preferred solution to address the complicated folding issues of the recombinant protein containing multiple disulfide bonds [27]. Indeed, the Takashi Yura group reported that the over-expression of bacterial protein disulfide isomerase (DsbC) and its modulator (DsbD) improved the production yield of horseradish peroxidase and human nerve growth factor, enzymes containing multiple disulfide bonds, in the periplasm of *E. coli* [28,29].

We report here the successful expression of the functional recombinant MBP-fused Omicron RBD using Dsb proteins-assisted periplasmic expression and the purification of the MBP-cleaved RBD by thrombin cleavage. The *E. coli*-expressing MBP-fused Omicron was well recognized by two anti-SARS-CoV-2-RBD antibodies (sotrovimab and CR3022) and showed a very similar human angiotensin-converting enzyme 2 (ACE2) binding affinity compared to mammalian cells expressing RBD. The expression system presented in this study will be a valuable tool to produce a recombinant spike RBD of subsequently emerging SARS-CoV-2 variants for developing a next variant-adapted vaccine or a neutralizing antibody.

## 2. Materials and Methods

### 2.1. Plasmid Construction

The expression vector of recombinant Omicron BA.1 RBDs was prepared by inserting the coding genes of the spike RBD (amino acids 333–530, which are numbered according to the definitions of the Centers for Disease Control and Prevention [29]; GenBank ID: UFO69279.1) with a His_6_ tag at the C-terminus with or without an MBP tag at the N-terminus. The coding genes were synthesized using a PCR-based gene synthesis method [30] and ligated into pET22b(+) using *Nco*I and *Bam*HI restriction endonuclease sites to construct the Omicron RBD expression vectors. The pET22b(+) vector was purchased from Novagen.

To develop an enzyme-assisted periplasmic expression system in *E. coli*, the duet vector for expressing Dsb proteins was prepared by inserting the coding genes of DsbC (amino acids 1–236; GenBank ID: HBL7408694.1) and DsbD (amino acids 77–565; GenBank ID: HAY4755864.1). The coding genes were also synthesized using a PCR-based gene synthesis method [30]. For inserting the coding gene of DsbC, we used *Nde*I and *Xho*I restriction enzymes after inserting the coding gene of DsbD at the *Nco*I and *Bam*HI sites of the pACYCduet-1. The pACYCduet-1 vector was purchased from Merck Millipore (Burlington, MA, USA). Hereafter, we defined the expression vector of Dsb proteins as a PSY (a co-expression system in periplasm with sufficient yield) vector.

To construct the genes of sotrovimab (S309, KEGG drug ID: D12014), VH and VL genes were synthesized by PCR assembly using the primers (Table S1), JS#324–JS#331 for VH and JS#333–JS#338 for VL. In addition, the VH (Genbank ID: DQ168569) and VL (Genbank ID: DQ168570) genes of CR3022 were synthesized using oPools™ Oligo Pools service (Integrated DNA Technologies Inc., Coralville, IA, USA). The variable regions of sotrovimab and CR3022 were PCR-amplified and assembled with the constant regions of the H- and the L-chains of human IgG1 [31] that were PCR amplified using pairs of primers (APEL#1 and APEL#2 for the H-chain; APEL#3 and APEL#4 for the L-chain, Table S1). The resulting PCR products were ligated into the pMAZ-IgL-GlycoT [31] using the *Bss*HII and *Xba*I sites for expression in mammalian cells. We confirmed the sequences of the coding genes prepared in this study by Sanger sequencing (Cosomogenetech, Korea)

### 2.2. Recombinant Proteins Expressed in Mammalian Cells

The spike RBDs of SARS-CoV-2 wild type or Omicron with a polyhistidine (His_6_) tag at the C-terminus were purchased from Sino Biological. The purified human Fc fusion ACE2 (hACE2-Fc) was prepared as described previously [32].

To express sotrovimab (S309) and CR3022, Expi293F cells (2 × 10^6^ cells/mL) were transfected with 300 μg of each plasmid encoding sotrovimab and CR3022 using 2400 μg of polyethylenimine (Polysciences, Taipei, Taiwan). After growing transfected cells for 6 days in the Gibco^TM^ FreeStyle^TM^ 293 Expression Medium (Thermo Fisher Scientific Inc., Waltham, MA, USA), the culture supernatants were mixed with 12.5 mL of 25× PBS and filtered through a 0.2 μm bottle top filter (Thermo Fisher Scientific Inc., Waltham, MA, USA). Then, the filtrates were incubated with 1 mL of Protein A resin (Puriogen, Yeosu, Korea) overnight at 4 °C. Following the transfer of the resins into a polypropylene column, 25 mL of 1× PBS and 3 mL of glycine-HCl buffer (100 mM, pH 2.7) were added to the column for washing and elution, respectively. After neutralizing the eluates by the addition of 1 mL of Tris-HCl buffer (1 M, pH 8.0) and exchanging the buffers with 1× PBS, the purified sotrovimab and CR3022 were concentrated using Amicon Ultra-4 spin columns (Merck Millipore). Trastuzumab (anti-HER2) was expressed and purified as described previously [33].

### 2.3. Expression and Purification of Omicron RBDs in E. coli

The Omicron RBD expression vectors were transformed into the *E. coli* strain BL21 Star^TM^ (DE3) with or without the PSY vector. BL21 Star^TM^ (DE3) was purchased from ThermoFisher. The transformed cells were grown in the Terrific Broth medium supplemented with 100 μg/mL of ampicillin and 30 μg/mL of chloramphenicol at 37 °C. The expression of the Omicron RBDs was induced by the addition of 0.2 mM isopropyl-β-D-1-thiogalactopyranoside (IPTG) for 18 h at 20 °C until the optical density of the cultured cells at 600 nm reached approximately 1.0. After harvesting the cultured cells by centrifugation, the cells were lysed by osmotic shock to isolate the periplasmic fraction. Briefly, the harvested cells were resuspended in 20 mL of chilled TES buffer (50 mM Tris-HCl, 1 mM EDTA, 20% sucrose, pH 8.0) per 1 L of cells. After incubation for 10 min on ice, the suspension in TES buffer was centrifuged (8000× *g*, 4 °C, 20 min), and the cell pellets were resuspended in 30 mL of 10 mM Tris-HCl pH 8.0. After incubation for 10 min on ice, the suspension in 10 mM Tris-HCl was centrifuged (12,000× *g*, 4 °C, 20 min). The supernatants from the first and second centrifugation cycles of osmotic shock were mixed and loaded onto a Ni-NTA agarose column (Qiagen) to purify the Omicron RBDs. The column was washed with 10 resin volumes of wash buffer (50 mM Tris-HCl, pH 7.5) and Omicron RBDs were eluted from the column using elution buffer (50 mM Tris-HCl, 300 mM imidazole, pH 7.5). The Ni-NTA eluates of the MBP-fused omicron RBDs were loaded onto Superdex 200 10/300 increase columns (Cytiva, Marlborough, MA, USA) pre-equilibrated in 1× PBS. The purification was verified by bis-tris sodium dodecyl-sulfate polyacrylamide gel electrophoresis (SDS-PAGE).

### 2.4. MBP Cleavage of MBP-Fused Omicron RBD by Thrombin

A lyophilized powder of bovine plasma thrombin (Sigma) was diluted in 1× PBS to a concentration of 1000 NIH units/mL, and 50 units of the prepared thrombin were added into the 1 mg of purified MBP-fused Omicron RBD and control samples. After incubation for 18 h at 25 °C, the mixture was loaded onto a Ni-NTA agarose column to separate the MBP-cleaved Omicron RBD. Then, the column was sequentially washed with 10 resin volumes of wash buffer 1 (50 mM Tris-HCl, pH 7.5) and wash buffer 2 (50 mM Tris-HCl, 5 mM imidazole, pH 7.5), and the MBP-cleaved Omicron RBDs were eluted from the column using an elution buffer (50 mM Tris-HCl, 300 mM imidazole, pH 7.5). The cleavage efficiency and purity of the Omicron RBDs were analyzed by bis-tris SDS-PAGE.

### 2.5. Bio-Layer Interferometry (BLI) Analysis

BLI was performed on an Octet^®^ BLI system R8 (Sartorius, Göttingen, Germany). A 10 mg/mL sample of hACE2-Fc was immobilized on an anti-human Fc-coated biosensor surface for 300 sec. After obtaining the baseline interference phase using 1× PBS, the sensors were subjected to association phase immersion in a sample plate containing the serial-diluted spike RBDs for 300 sec. Then, the sensors were immersed in 1× PBS for 300 s. The mean k_on_, k_off_, and K_D_ values were calculated using Octet BLI Analysis 12.2 software (Sartorius, Göttingen, Germany), and all binding curves based on their global fit to a Langmuir 1:1 binding model showed an R^2^ value greater than 0.99.

### 2.6. Enzyme-Linked Immunosorben Assay (ELISA)

To perform an enzyme-linked immunosorbent assay (ELISA), 4 μg of the recombinant RBDs, MBP, or an extracellular domain of HER-2 was immobilized on the high-binding plate for 2 h at room temperature. After blocking using 4% skim milk, the plate was washed with 1× PBS (pH 7.4) containing 0.05% Tween 20 (PBST), and a serial-diluted antibody (sotrovimab, CR3022, or trastuzumab) in PBST was added. To develop colorimetric binding signals, a Protein A-HRP conjugate (GenScript, Piscataway, NJ, USA) was added after washing the plate. The colorimetric signals were measured at 450 nm by an Epoch Microplate reader (Biotek, Winooski, VT, USA).

## 3. Results

### 3.1. Expression and Purification of Soluble Omicron RBDs

**Figure 1:**
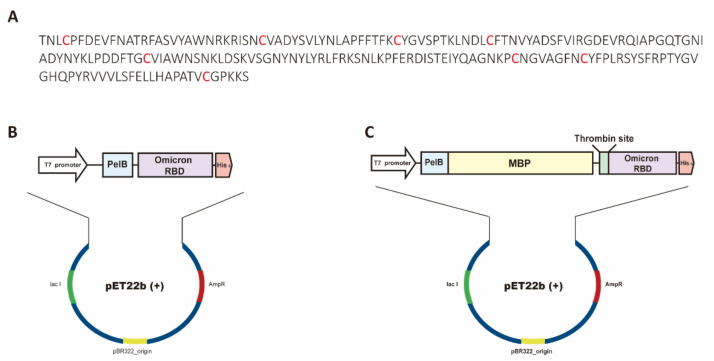
Construction of RBD_omi_ and MBP-RBD_omi_ vectors. (**A**) Amino acid sequence of RBD_omi_ used in this study. The red letters indicate cysteine residues that form disulfide bonds. (**B**,**C**) Vector maps of (**B**) RBD_omi_ and (**C**) MBP-RBD_omi_ vectors. The schematic domains are colored as follows: T7 promoter (white), PelB signal sequence (sky blue), MBP (yellow), thrombin recognition site (green), RBDomi (purple), and His6 tag (light red).

The coding genes for the spike RBD of SARS-CoV-2 Omicron (Figure 1A) with or without the MBP fusion tag were synthesized and codon-optimized by the PCR-based gene synthesis method [30]. Hereafter, we call the recombinant protein of the Omicron RBD RBD_omi_ and that of the Omicron RBD with the MBP tag, MBP-RBD_omi_. All coding genes included a His_6_ tag after the RBD_omi_ sequence (Figure 1B,C), and the MBP-RBD_omi_ gene contained a thrombin recognition site downstream of the MBP sequence (Figure 1C). The coding genes were cloned into pET22b(+) to secrete the recombinant proteins from the cytoplasm to the periplasm of *E. coli* (Figure 1B,C). To express the recombinant proteins, MBP-RBD_omi_ and RBD_omi_ vectors were transformed or co-transformed with the PSY vector, the expression vector for Dsb proteins (Figure S1), into BL21 Star^TM^ (DE3).

**Figure 2:**
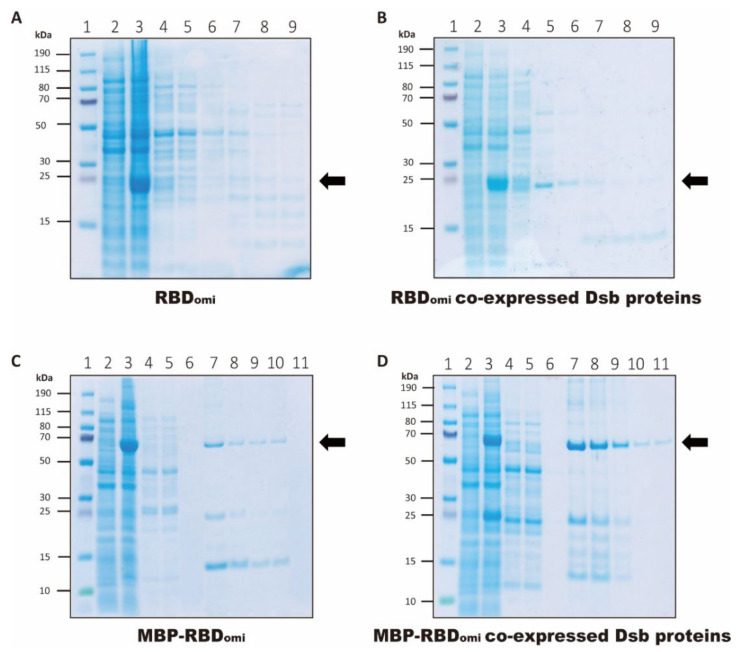
Expression and Ni-NTA purification of RBD_omi_ and MBP-RBD_omi_. Purification of (**A**) RBD_omi_, (**B**) RBD_omi_ co-expressed Dsb proteins, (**C**) MBP-RBD_omi_, and (**D**) MBP-RBD_omi_ co-expressed Dsb proteins was analyzed by bis-tris SDS-PAGE. Black arrows indicate the band positions of the recombinant proteins. The lanes of SDS-PAGE are as follows: lane 1, protein size marker (Thermo); lane 2, cell lysate before IPTG induction; lane 3, cell lysate after IPTG induction; lane 4, periplasmic fraction obtained by osmotic shock; lane 5, Ni-NTA flow through fraction; lane 6, Ni-NTA wash fraction; lane 7 to 11, Ni-NTA elution fractions.

To determine an optimal cultivation condition for the co-expression of MBP-RBD_omi_ and Dsb proteins, we attempted to culture transformants under three IPTG concentrations (0.2 mM, 0.5 mM, and 1 mM) and two incubation temperatures (20 °C and 30 °C). The results showed that the expression level of recombinant MBP-RBD_omi_ at 20 °C was higher than that at 30 °C (Figure S2), and the IPTG concentration did not exert a significant effect on the yield of recombinant MBP-RBD_omi_ (Figure S2). Therefore, we induced the expression of MBP-RBD_omi_ or RBD_omi_ by adding 0.2 mM of IPTG and incubating for 18 h at 20 °C. Although a high expression of MBP-RBD_omi_ or RBD_omi_ in the cell lysates after induction was observed under all expression conditions in this study (Figure 2), a high secretion of MBP-RBD_omi_ or RBD_omi_ in the periplasmic fraction was observed in the Coomassie blue-stained SDS-PAGE only when using the PSY that allowed the co-expression of Dsb proteins (Figure 2B,D, lane 4). These results indicate that Dsb proteins improved the production yield of recombinant proteins in the periplasm of *E. coli*.

After Ni-NTA purification, MBP-RBD_omi_ or RBD_omi_ were observed at the respective ~66 kDa or ~24 kDa band in the Coomassie blue-stained SDS-PAGE, except in the RBD_omi_-only expression condition (Figure 2). Interestingly, we also observed proteins with a molecular weight (MW) less than 24 kDa under all expression conditions (Figure 2). Because the spike RBD of Omicron has a lower melting temperature and a higher protease digestion sensitivity than those of SARS-CoV-2 wild type [34], the proteins with a MW less than 24 kDa likely represent degradation products of RBD_omi_.

**Figure 3:**
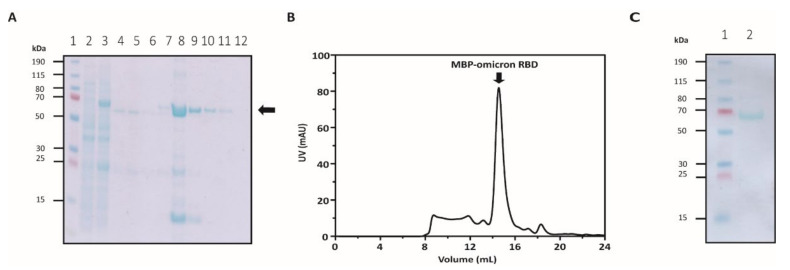
Purification of MBP-RBD_omi_. (**A**) SDS-PAGE analysis of purified MBP-RBD_omi_ by Ni-NTA. The black arrow indicates the band position of the MBP-RBD_omi_. The lanes of SDS-PAGE are as follows: lane 1, protein size marker (Thermo); lane 2, cell lysate before IPTG induction; lane 3, cell lysate after IPTG induction; lane 4, periplasmic fraction obtained by osmotic shock; lane 5, Ni-NTA flow through fraction; lane 6, Ni-NTA wash fraction; lane 7 to 12, Ni-NTA elution fractions. (**B**) Size-exclusion spectrum of MBP-RBD_omi_ monitored at 280 nm. The black arrow indicates the position of MBP-RBD_omi_ fractions. (**C**) SDS-PAGE analysis of purified MBP-RBD_omi_ by size-exclusion. The lanes of SDS-PAGE are as follows: lane 1, protein size marker (Thermo); lane 2, purified MBP-RBD_omi_.

**Figure 4:**
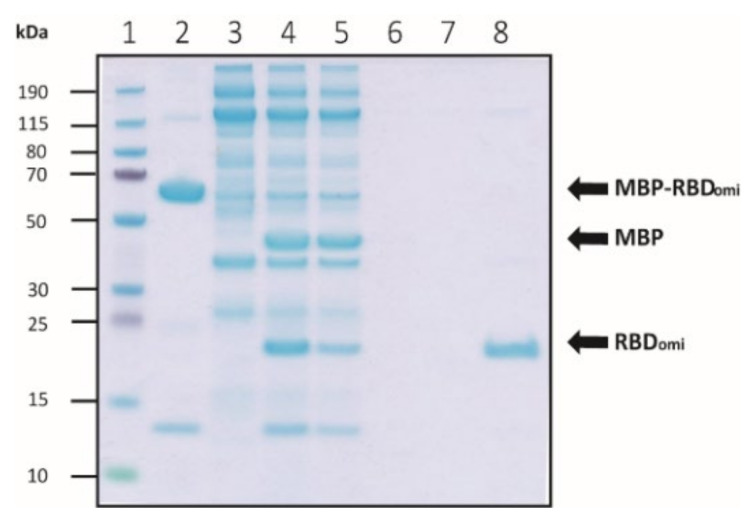
Purification of the soluble MBP-cleaved RBD_omi_. Purification and cleavage efficiency were analyzed by bis-tris SDS-PAGE. The lanes of SDS-PAGE are as follows: lane 1, protein size marker (Thermo); lane 2, purified MBP-RBD_omi_; lane 3, 50 units of thrombin; lane 4, thrombin digested MBP-RBD_omi_; lane 5, Ni-NTA flow through fraction; lane 6, Ni-NTA first wash fraction; lane 7, Ni-NTA second wash fraction; lane 8, Ni-NTA elution fraction.

The highest yield of the recombinant protein was observed under the co-expression of MBP-RBD_omi_ and PSY vectors (Figure 2D and Figure 3A), indicating that MBP fusion and Dsb protein-assisted periplasmic expression could solve the complicated folding problem of the recombinant spike RBD containing multiple disulfide bonds (Figure 1A). After further purification by size-exclusion chromatography, approximately 0.1 mg of pure MBP-RBD_omi_ expressed by the Dsb proteins-assisted periplasmic expression system was obtained from 1 L of flask culture (Figure 3B,C). Additionally, the soluble untagged RBD_omi_ was obtained by thrombin cleavage of the MBP fusion tag and was isolated with 80% recovery by Ni-NTA (Figure 4). The remaining 20% of the MBP-cleaved RBD_omi_ did not bind to Ni-NTA (Figure 4, lane 5), which would be a degradation of the C-terminal His_6_-tag.

### 3.2. E. coli-Expresed MBP-RBD_omicron_ Exhibits Selevtive Binding to Sotrovimab, CR3022, and Human ACE2

**Figure 5:**
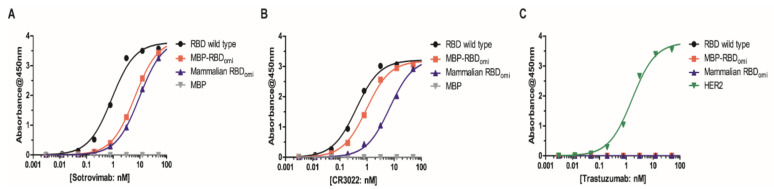
ELISA binding assay of *E. coli*-expressed MBP-RBD_omi_ and mammalian cell-expressed RBDs to (**A**) sotrovimab, (**B**) CR3022, and (**C**) trastuzumab. MBP and HER2 were used as controls in this study. The black, red, blue, gray, and green dots indicate the absorbances of RBD wild type, MBP-RBD_omi_, mammalian cell-expressing RBD_omi_, MBP, and HER2, respectively. Error bars are ±standard deviation of triplicate experiments.

To examine whether the *E. coli*-expressed MBP-RBD_omi_ was specifically recognized by human antibodies with high binding affinity to the SARS-CoV-2 RBD, we conducted ELISA assays using two previously reported human SARS-CoV-2 antibodies (sotrovimab and CR3022) and the trastuzumab antibody as a negative control. Previous studies have reported that both sotrovimab (an approved neutralizing antibody for the treatment of Omicron BA.1 infection [35]) and CR3022 broadly bound to the RBDs of SARS-CoV-2 wild type and its variants including Omicron BA.1 [36]. In addition, the binding affinities of sotrovimab and CR3022 to RBD_omi_ were lower than those to RBD wild type [36]. Our purified sortrovimab and CR3022 also showed proper binding properties to RBD_omi_ and RBD wild type (Figure 5A,B). However, neither RBD_omi_ nor RBD wild type bound to the unassociated antibody, trastuzumab (anti-HER2 antibody) (Figure 5C). The results of ELISA revealed an association of sotrovimab and CR3022 with MBP-RBD_omi_ as well as mammalian expressed RBDs of SARS-CoV-2 wild type and Omicron (Figure 5A,B). On the other hand, trastuzumab (anti-HER2 antibody) showed no binding to either MBP-RBD_omi_ or mammalian expressed RBDs as expected (Figure 5C). Taken together, these results indicate that *E. coli*-expressing MBP-RBD_omi_ is highly functional.

**Figure 6:**
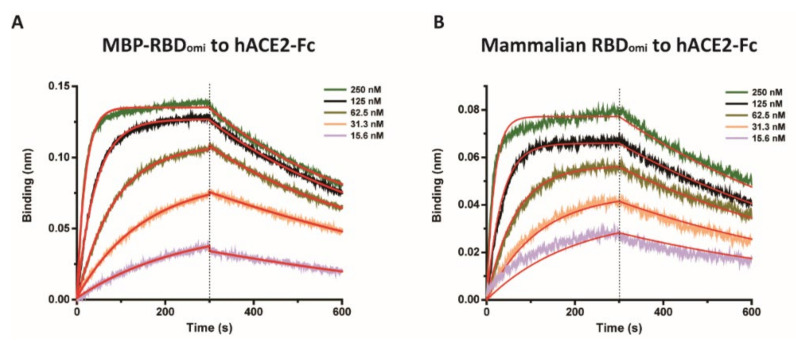
Binding assay of (**A**) MBP-RBD_omi_ and (**B**) RBD_omi_ expressed in a mammalian system to hACE2-Fc determined by BLI. Data are shown as colored lines at different concentrations of recombinant RBDs. Red lines are the best fits of the data.

To confirm the biochemical activity of MBP-RBD_omi_, the binding affinity of MBP-RBD_omi_ to the human ACE2 receptor (hACE2) was examined using BLI (Figure 6) after confirming the binding of purified hACE2-Fc to the recombinant RBD wild type expressed in mammalian cells by enzyme-linked immunosorbent assay (Figure S3). The kinetic parameters of MBP-RBD_omi_ are similar to those of the omicron RBD expressed in a mammalian system (Table 1), suggesting that *N*-linked glycans appended on N331 and N343 of the RBD expressed in mammalian cells do not exert a significant effect on human ACE2 binding.

## 4. Discussion

In this study, we used a disulfide isomerase-assisted periplasmic expression system to solve the folding problems of a bacterial expression system and successfully produced a soluble and pure spike RBD of the SARS-CoV-2 Omicron in *E. coli*. Although the final yield of MBP-RBD_omi_ was low, the reason might not be the Dsb proteins-assisted periplasmic expression system. More than 2 mg of the pure MBP-fused RBD wild type was expressed by the Dsb proteins-assisted periplasmic expression system from 1 L of shake flask culture (Figure S4). In addition, the final yield of MBP-RBD wild type expressed by the CyDisCo system, the enzyme (Evr1p and PDI)-assisted cytoplasmic expression system, was 0.25 mg from 1L of flask culture [24]. Based on these findings, it is highly likely that the low yield of MBP-RBD_omi_ can be attributed to the increased instability of RBD_omi_ compared to the that of RBD wild type [26,34]. Therefore, purifying the MBP-RBD_omi_ as quickly as possible at a low temperature would be highly desirable to improve the final yield of the protein.

In this study, we confirmed that aglycosylated SARS-CoV-2 RBDs could be rapidly prepared through shake flask bacterial culture in complex media (Terrific Broth). A bacterial periplasmic expression system enables high-cell density cultivation through fed-batch fermentation in defined minimal media at low cost, and it is easy to scale-up for the production of a large amount of various complex recombinant proteins, including aglycosylated full-length IgG antibodies [31,37]. Therefore, if the promoter and 5’-untranslated region (5’-UTR) sequence is optimized, the expression level is evaluated in more diverse *E. coli* strains, and the cultivation parameters are optimized in subsequent studies, the yield of MBP-RBD_omi_ will be improved.

The recombinant RBDs of SARS-CoV-2 wild type and its variants have two *N*-glycosylation sites (N331 and N343) [38]. Although the enzyme-assisted periplasmic expression system in *E. coli* could not produce a glycosylated RBD, the binding affinity of MBP-RBD_omi_ to hACE2 was highly similar to that of the recombinant RBD_omi_ expressed in the mammalian system. Moreover, MBP-RBD_omi_ could also interact with the SARS-CoV-2 antibodies sotrovimab and CR3022. These results indicate that *E. coli* expressing MBP-RBD_omi_ has proper physicochemical activity and the MBP tag did not interrupt the interaction between RBD_omi_ and its binding partners. Therefore, the bacterial system in this study could be suitable to produce a functional and correctly folded spike RBD of future SARS-CoV-2 variants promptly at low cost. Aglycosylated or deglycosylated antigen has high potential to be used for the development of broadly neutralizing antibodies against an infectious disease because removing the glycan appended on the antigen could expose the conserved peptide epitopes [39].

It is notable that the aglycosylated RBD increased the sensitivity to RBD binding antibodies [40] and the subunit vaccine candidate based on the non-glycosylated SARS-CoV-2 RBD induced neutralizing antibodies against SARS-CoV-2 wild type, Delta, and Omicron variants [41]. Our ELISA results also showed that the binding affinity of MBP-RBD_omi_ to sotrovimab or CR3022 was higher than that of mammalian-expressing RBD_omi_ (Figure 5A,B). In this study, we could isolate aglycosylated and MBP-cleaved RBD_omi_ by thrombin cleavage. Therefore, the Dsb proteins-assisted periplasmic expression system could be the basis of a new subunit vaccine for eliciting a neutralizing antibody against the next variant of SARS-CoV-2.

## Figures and Tables

**Table 1:** Kinetic constants for the binding affinities of the Omicron RBDs to the human ACE2 receptor determined by BLI.

	**k_on_ (1/M · s)**	**k_off_ (1/s)**	**K_D_ (M)**
**MBP-RBD_omi_**	1.72 × 10^5^	1.74 × 10^−3^	10.1 × 10^−9^ ± 0.06 × 10^−9^
**Mammalian RBD_omi_**	2.26 × 10^5^	1.61 × 10^−3^	7.16 × 10^−9^ ± 0.07 × 10^−9^

## Data Availability

Not applicable.

## Supplementary Materials

The following supporting information can be downloaded at: https://www.mdpi.com/article/10.3390/bioengineering9110670/s1, Table S1: Primers used for cloning of sotrovimab and CR3022; Figure S1: Construction of the PSY vector; Figure S2: Expression of MBP-RBD_omi_ in various culture conditions; Figure S3: ELISA binding assay showing the affinity of hACE2-Fc for recombinant RBD of SARS-CoV-2 wild type expressed in a mammalian system; Figure S4: Ni-NTA purification of MBP-RBD wild type expressed by the Dsb proteins-assisted periplasmic expression system.
