# Supplementary material for: Functional Expression of the Recombinant Spike Receptor Binding Domain of SARS-CoV-2 Omicron in the Periplasm of Escherichia coli
Source: Bioengineering (Basel). 2022 Nov 10;9(11):670. doi: 10.3390/bioengineering9110670 (PMC9687980; doi:10.3390/bioengineering9110670)
Supplement: Supplementary file 1 [file bioengineering-09-00670-s001.zip › bioengineering-1926115-supplementary.pdf]

Table S1. Primers used for cloning of sotrovimab and CR3022

Primer name	Primer nucleotide sequence (5' ➔ 3')
JS#324	CGC AGC GAG CGC GCA CTC CCA GGT GCA GCT GGT GCA GAG CGG AGC GGA A
JS#325	GGG GTA GCC GCT GGC CTT GCA GCT CAC CTT CAC GCT GGC ACC GGG CTT CTT CAC TTC CGC TCC GCT CTG C
JS#326	GCC AGC GGC TAC CCC TTC ACC TCC TAC GGC ATC AGC TGG GTG AGG CAG GCC CCA GGA CAA GGT CTG GAG T
JS#327	ACT TCT GGG CGT AGT TGG TGT TTC CCT GGT AGG TGC TGA TCC AGC CCA TCC ACT CCA GAC CTT GTC CTG G
JS#328	ACC AAC TAC GCC CAG AAG TTC CAA GGC AGG GTG ACC ATG ACC ACC GAC ACC AGC ACC ACC ACC GGC TAC A
JS#329	CCT GGC GCA GTA GTA CAC GGC GGT GTC GTC GCT CCT CAG CCT CCT GAG CTC CAT GTA GCC GGT GGT GGT G
JS#330	GTG TAC TAC TGC GCC AGG GAC TAC ACC AGG GGC GCA TGG TTC GGC GAG AGC CTG ATC GGC GGC TTC GAC A
JS#331	GAT GGG CCC TTG GTG CTA GCG CTG CTC ACG GTA ACC AGT GTT CCC TGT CCC CAG TTG TCG AAG CCG CCG AT
JS#333	CGC AGC GAG CGC GCA CTC CGA GAT CGT TCT GAC CCA GAG CCC CGG AAC ACT GAG CCT GAG TCC CGG CGA ACG CGC CAC GC
JS#334	CTG TTG ATA CCA GGC AAG AGA GGT GCT GCT CAC GGT CTG GCT GGC CCT GCA GCT AAG CGT GGC GCG TTC G
JS#335	TCT CTT GCC TGG TAT CAA CAG AAG CCA GGC CAA GCC CCC AGG CTG CTG ATC TAC GGC GCC AGC AGT AGG G
JS#336	GCG TGA AGT CGG TGC CGC TCC CGC TGC CGC TGA ACC TGT CGG GGA TGC CGG TGG CCC TAC TGC TGG CGC C
JS#337	GGC ACC GAC TTC ACG CTG ACC ATC TCA AGG CTG GAG CCC GAG GAC TTC GCC GTG TAC TAC TGC CAA CAG C
JS#338	CCT CTT GAT CTC CAC CTT AGT GCC GCC ACC AAA GGT CAG GCT GGT GTC ATG CTG TTG GCA GTA GTA CAC G
APEL#1	GCT AGC ACC AAG GGC CCA TC
APEL#2	GAA TTC CGC TCT AGA TTA TCA TTT ACC CGG AGA CAG GGA GAG GC
APEL#3	ACG GTG GCC GCA CCA TC
APEL#4	GAA TTC CGC TCT AGA TTA TCA GCA CTC TCC CCT GTT GAA GCT C

A

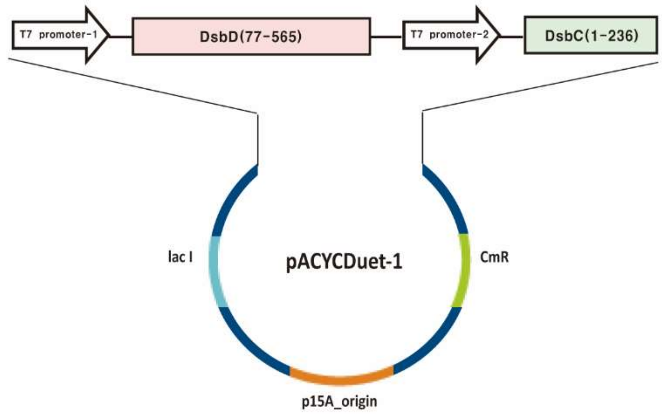

B

MVQLPQGVWHEDEFYGKSEIYRDRLTLPVTINQASAGATLTVTYQGCADAGFCYPPETKTVPLSEVVANNAAPQPVS
VPQQEQPTAQLPFSALWALLIGIGIAFTPCVLPMYPLISGIVLGGKQRLSTARALLLTFIYVQGMALTYTALGLVVAAAGL
QFQAALQHPYVLIGLAIVFTLLAMSMFGLFTLQLPSSLQTRLTLMSNRQQGGSPGGVFVMGAIVGLICSPCTTAPLSA
ILLYIAQSGNMWLGGGTLYLYALGMGLPLMLITVFGNRLLPKSGPWMEQVKTAFGFVILALPVFLLERVIGDVWGLRL
WSALGVAFFGWAFITSLQAKRGWMRIVQIILLAAALVSVRPLQDWAFGATHTAQTQTHLNFTQIKTVDELNQALVEA
KGKPVMLDLYADWCVACKEFEKYTFSDPQVQKALADTVLLQANVTANDAQDVALLKHLNVLGLPTILFFDGQGQEH
PQARVTGFMDAETFSAHLRDRQP

C

MKKGFMLFTLLAAFSGFAQADDAAIQQTLAKMGIKSSDIQPAPVAGMKTVLTNSGVLYITDDGKHIIQGPMYDVSGT
APVNVTNKMLLKQLNALEKEMIVYKAPQEKHVITVFTDITCGYCHKLHEQMADYNALGITVRYLAFPRQGLDSDAEK
EMKAIWCAKDKNKAFDDVMAGKSVAPASCDVDIADHYALGVQLGVSGTPAVVLSNGTLVPGYQPPKEMKEFLDEH
QKMTSGK

Figure S1. Construction of the PSY vector. (**A**) Vector map of the expression of DsbC and DsbD. The schematic domains are colored as follows: T7 promoters (white), DsbD (light red), and DsbC (light green). (**B**,**C**) Amino acid sequences of (**B**) DsbD and (**C**) DsbC.

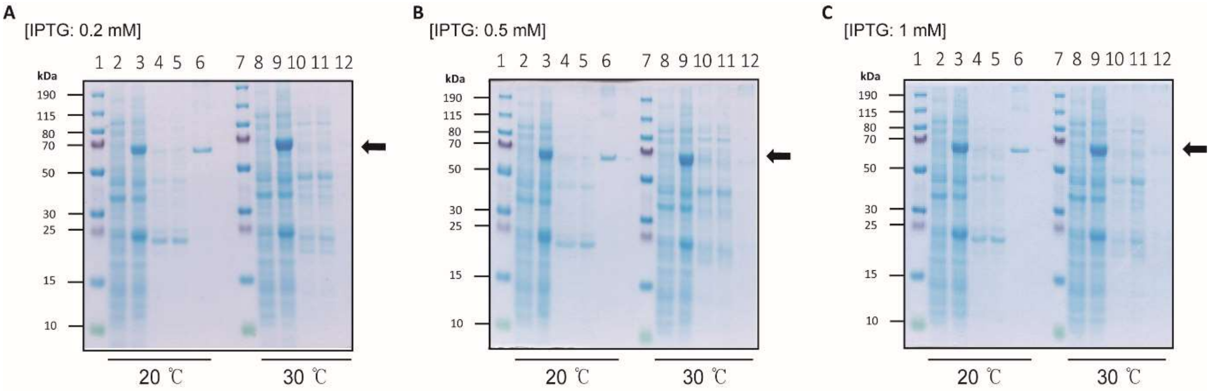

Figure S2. Expression of MBP-RBD_{omi} in various culture conditions. MBP-RBD_{omi} was expressed by the Dsb proteins-assisted periplasmic expression system. The expression of RBD_{omi} was induced by addition of (**A**) 0.2 mM, (**B**) 0.5 mM, or (**C**) 1 mM IPTG for 18 hours at 20 °C or 30 °C. After expression, the cultured cells were lysed by osmotic shock to isolate the periplasmic fraction. The periplasmic fraction was loaded onto a Ni-NTA agarose column to purify MBP-RBD_{omi}. The purification was verified by bis-tris SDS-PAGE. Black arrows indicate the band positions of the MBP-RBD_{omi}. The lanes of SDS-PAGE are as follows: lane 1 and 7, protein size marker (Thermo); lane 2 and 8, cell lysate before IPTG induction; lane 3 and 9, cell lysate after IPTG induction; lane 4 and 10, periplasmic fraction obtained by osmotic shock; lane 5 and 11, Ni-NTA flow through fraction; lane 6 and 12, Ni-NTA elution fraction.

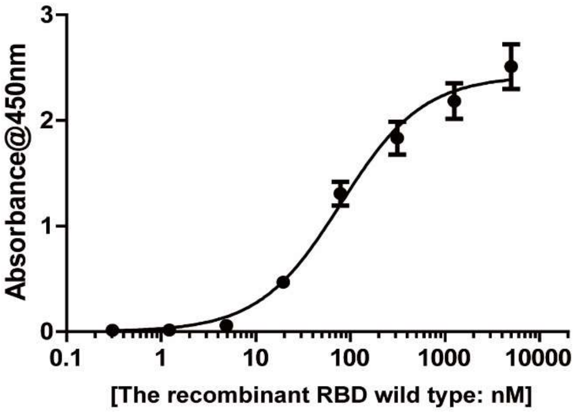

Figure S3. ELISA binding assay showing the affinity of hACE2-Fc for recombinant RBD of SARS-CoV-2 wild type expressed in a mammalian system. For that, 2 µg of hACE2-Fc was immobilized on the high-binding plate for 2 hours at room temperature. After blocking using 5% skim milk, the plate was washed with 1× PBS (pH 7.4) containing 0.1% Triton X-100 (PBST), and the serial-diluted recombinant RBD in PBST was added. To develop colorimetric signals of the binding affinity, anti-His_6 tag-HRP conjugate (Sigma) was added after washing the plate. The colorimetric signals were monitored at 450 nm by an Infinite M200 pro (Tecan). Error bars are ±standard deviation of triplicate experiments.

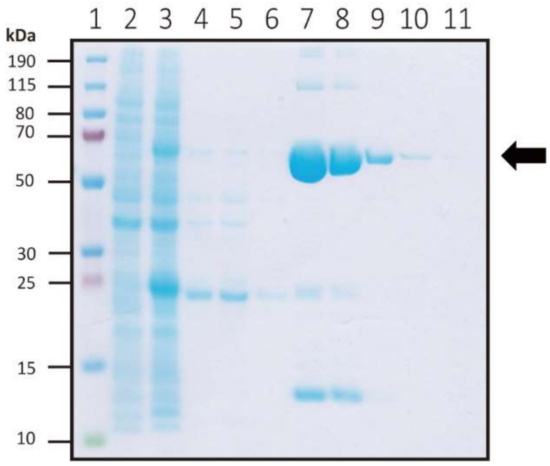

Figure S4. Ni-NTA purification of MBP-RBD wild type expressed by the Dsb proteins-assisted periplasmic expression system. Purification was verified by bis-tris SDS-PAGE. The black arrow indicates the band position of the MBP-RBD wild type. The lanes of SDS-PAGE are as follows: lane 1, protein size marker (Thermo); lane 2, cell lysate before IPTG induction; lane 3, cell lysate after IPTG induction; lane 4, periplasmic fraction obtained by osmotic shock; lane 5, Ni-NTA flow through fraction; lane 6, Ni-NTA wash fraction; lane 7 to 11, Ni-NTA elution fractions.
